# Distinct clinical and neuropathological features of G51D *SNCA* mutation cases compared with *SNCA* duplication and H50Q mutation

**DOI:** 10.1186/s13024-015-0038-3

**Published:** 2015-08-27

**Authors:** Aoife P. Kiely, Helen Ling, Yasmine T. Asi, Eleanna Kara, Christos Proukakis, Anthony H. Schapira, Huw R. Morris, Helen C. Roberts, Steven Lubbe, Patricia Limousin, Patrick A. Lewis, Andrew J. Lees, Niall Quinn, John Hardy, Seth Love, Tamas Revesz, Henry Houlden, Janice L. Holton

**Affiliations:** Department of Molecular Neuroscience, Queen Square Brain Bank, UCL Institute of Neurology, Queen Square, WC1N 3BG London, UK; Department of Molecular Neuroscience and Reta Lila Weston Institute of Neurological Studies, UCL Institute of Neurology, London, UK; Sobell Department of Motor Neuroscience and Movement Disorders, Unit of Functional Neurosurgery, UCL Institute of Neurology, UCL, London, UK; School of Pharmacy, University of Reading, Whiteknights, Reading, UK; Department of Clinical Neuroscience, UCL Institute of Neurology, London, UK; Academic Geriatric Medicine, University of Southampton, Southampton, UK; National Hospital for Neurology and Neurosurgery, Queen Square, London, UK; Clinical Neurosciences, University of Bristol, Bristol, UK; Alzheimer’s Disease Research Centre, Harvard medical school & Massachusetts General Hospital, 114 16th Street, Charlestown, MA 02129 USA

**Keywords:** Parkinson’s disease, Multiple system atrophy, α-synuclein, Clinical features, Phosphorylation, Mutation, *SNCA*

## Abstract

**Background:**

We and others have described the neurodegenerative disorder caused by G51D *SNCA* mutation which shares characteristics of Parkinson’s disease (PD) and multiple system atrophy (MSA). The objective of this investigation was to extend the description of the clinical and neuropathological hallmarks of G51D mutant *SNCA*-associated disease by the study of two additional cases from a further G51D *SNCA* kindred and to compare the features of this group with a *SNCA* duplication case and a H50Q *SNCA* mutation case.

**Results:**

All three G51D patients were clinically characterised by parkinsonism, dementia, visual hallucinations, autonomic dysfunction and pyramidal signs with variable age at disease onset and levodopa response. The H50Q *SNCA* mutation case had a clinical picture that mimicked late-onset idiopathic PD with a good and sustained levodopa response. The *SNCA* duplication case presented with a clinical phenotype of frontotemporal dementia with marked behavioural changes, pyramidal signs, postural hypotension and transiently levodopa responsive parkinsonism. Detailed post-mortem neuropathological analysis was performed in all cases. All three G51D cases had abundant α-synuclein pathology with characteristics of both PD and MSA. These included widespread cortical and subcortical neuronal α-synuclein inclusions together with small numbers of inclusions resembling glial cytoplasmic inclusions (GCIs) in oligodendrocytes. In contrast the H50Q and *SNCA* duplication cases, had α-synuclein pathology resembling idiopathic PD without GCIs. Phosphorylated α-synuclein was present in all inclusions types in G51D cases but was more restricted in *SNCA* duplication and H50Q mutation. Inclusions were also immunoreactive for the 5G4 antibody indicating their highly aggregated and likely fibrillar state.

**Conclusions:**

Our characterisation of the clinical and neuropathological features of the present small series of G51D *SNCA* mutation cases should aid the recognition of this clinico-pathological entity. The neuropathological features of these cases consistently share characteristics of PD and MSA and are distinct from PD patients carrying the H50Q or *SNCA* duplication.

**Electronic supplementary material:**

The online version of this article (doi:10.1186/s13024-015-0038-3) contains supplementary material, which is available to authorized users.

## Background

The *SNCA* gene encodes the α-synuclein protein, which has a predicted molecular weight of 17 kDa, is expressed abundantly in human brain and is believed to function in vesicle recycling [1]. α-Synucleinopathies, such as Parkinson’s disease (PD), multiple system atrophy (MSA) and dementia with Lewy bodies (DLB), share the pathological hallmark of intracellular inclusions in which α-synuclein is a major constituent. In PD and DLB the pathological α-synuclein inclusions are largely neuronal in the form of Lewy bodies (LB) and Lewy neurites (LN) while in MSA the most frequent site of aggregated α-synuclein is the oligodendrocyte forming glial cytoplasmic inclusions (GCIs). Previously, multiplications (duplications and triplications) as well as several missense point mutations of the *SNCA* gene: A53T [2], E46K [3], A30P [4] and H50Q [5], were found to cause autosomal dominant PD. We recently reported a novel G51D *SNCA* mutation [6], which resulted in clinical and neuropathological features with some similarities to both PD and MSA. Affected family members developed early-onset Parkinson’s disease with dementia. Neuropathological features included CA2-CA3 hippocampal and cortical neuronal loss with widespread, numerous neuronal α-synuclein positive cytoplasmic inclusions together with smaller numbers of oligodendroglial inclusions that resembled the GCIs of MSA and so were referred to as GCI-like inclusions. A similar combined PD and MSA profile was also recently reported in a Finnish family carrying a novel A53E mutation [7]. Known *SNCA* mutations cluster in a putative protein loop, disruption of which may significantly alter the behaviour of the α-synuclein protein by affecting lipid binding and fibril formation [8]. The neuropathological appearances associated with different mutations have varied considerably, but it is still unclear how alteration in α-synuclein structure determines the neuropathology. A genetic cause has not been demonstrated in MSA, although recessive *COQ2* mutations have been suggested to underlie a subset of familial MSA cases in the Japanese population [9].

Following our initial G51D mutation study, additional cases were reported in France [10] and Japan [11]. Affected family members in both reports had a similar clinical progression to that in our reported family, with early-onset levodopa-responsive parkinsonism accompanied by dementia. The clinical symptoms of the G51D mutation case described by Tokutake and colleagues closely resembled those in the proband of the family which we have reported [11]. Their case presented with levodopa-responsive parkinsonism at a young age (28 years), dementia, hallucinations and autonomic dysfunction. Lesage et al. noted widespread neuronal α-synuclein pathology, which was similar in distribution and morphology to that which we observed: however, they did not report GCI-like inclusions [10].

We have recently identified a second British family with the G51D α-synuclein mutation and have investigated post-mortem brain tissue from two affected family members. In the current study we sought to assess the spectrum and variability of clinical and neuropathological features in the three G51D cases (these two and our original case), and to determine whether there are particular phenotypic features that may suggest a G51D mutation and indicate that analysis of *SNCA* is required.

For comparison, we analysed the clinical and neuropathological features in a H50Q mutation case and in a *SNCA* duplication case: some details of each of these cases have been published previously [5, 12].

We consider both the H50Q mutation case and the *SNCA* duplication case to be pertinent comparisons with the G51D cases. The H50Q mutation site is immediately adjacent to the G51D site within the region of α-synuclein which is important for fibril formation and lipid binding [8]. It might therefore be expected that there would be strong similarities between the phenotypical profiles of this case and the G51D mutation cases. We previously reported extensive α-synuclein pathology on post-mortem examination of a case with a large 6.4 Mb duplication of the *SNCA* locus [12]. This case was used as a further comparator as this previous report suggested that the duplication of *SNCA* can result in severe α-synuclein pathology resembling that of G51D mutation [6, 12].

We have demonstrated in this detailed clinical and neuropathological study that G51D mutation cases share a constellation of features of parkinsonism with dementia, visual hallucination and autonomic dysfunction, with abundant α-synuclein pathology with characteristics of both PD and MSA. The G51D mutation cases differ from the late-onset, relatively benign, PD-like presentation in the case with a H50Q mutation but share similarities with the *SNCA* duplication case. However, the neuropathological features of the G51D cases were distinct from those of H50Q mutation and *SNCA* duplication, although there were some common findings when the post-translational modifications of α-synuclein were explored.

## Results

The clinical features of all cases are summarised in Table 1.

**Table 1:** Summary of clinical findings

Case	Case one (G51D)	Case two (G51D)	Case three (G51D)	H50Q	Duplication
Age of onset (years)	19	69	46	71	38
Disease duration (years)	29	6	6	12	12
Presenting symptoms	Resting hand tremor, anxiety	Resting hand tremor, anxiety and depression	Resting hand tremor, depression	Resting hand tremor	Resting hand tremor and tongue tremor
Final clinical diagnosis	Familial pallidopyramidal syndrome	Parkinson’s disease with dementia	Parkinson’s disease with dementia	Parkinson’s disease	FTDP-17
Levodopa responsive	Good and sustained	Transient	Transient	Good and sustained	Transient
Motor fluctuation	Yes	No	No	No	No
Dystonia	Wearing-off foot dystonia	Blepharospasm (unrelated to dopamine replacement therapy)	No	Blepharospasm (unrelated to dopamine replacement therapy)	Blepharospasm and cervical dystonia (unrelated to dopamine replacement therapy)
Latency from first symptom of onset of cognitive impairment (years)	8	2	2	Not applicable	9
Prominent cognitive impairment	Yes	Yes	Yes	No	Yes
Predominant frontal cognitive impairment	No	Yes (emotional lability, apathy, disinhibition)	Yes (perseveration, frontal executive impairment, grasp reflex)	No	Yes (obsessive behaviour, self-neglect, markedly increased appetite, grasp reflex, perseveration, motor recklessness)
Visual hallucinations (unrelated to drug effect)	Yes	Yes	Yes	No	Yes
Autonomic dysfunction	Yes	Yes	Yes	No	Yes
Pyramidal signs (pathological reflexes and extensor plantar response)	Yes	Yes	Yes	No	Yes
Additional features	Myoclonus seizures	Vertical supranuclear gaze palsy, apraxia of eyelid opening	Not applicable	No	Not applicable
Family history of parkinsonism	Father, sister	Mother, Aunt, brother, Son	Mother, uncle, grandmother, great aunt	No	Father, male cousin, grandmother, two great aunts

### G51D case one: family one, patient II: 1 Fig. 1a

We have previously described the clinical history of case one [6]. The index case was a British Caucasian male who presented at age 19 with left hand tremor and slowly progressive, asymmetrical, levodopa-responsive parkinsonism with marked motor fluctuation. Cognitive decline and visual hallucination started 9 years after disease onset. Autonomic dysfunction, pyramidal signs, myoclonus and seizures were also noted. The disease duration was 29 years. His father who had a background of depression and obsessional personality, also developed parkinsonism at age 39 followed by dementia, and died of sepsis at age 47.

**Fig. 1:**
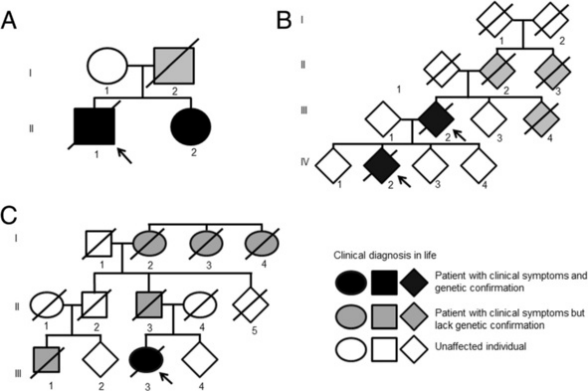
Genetic Pedigrees. Simplified pedigree structures in which arrows are used to indicate the proband, circles indicate females, squares indicate males and diamonds indicate individuals of indeterminate or undisclosed gender, **a** G51D case one (patient II,1). The father of case one was diagnosed with PD (*grey*), his mother was unaffected (*white*). His sister carries the G51D mutation and developed PD symptoms at 40 years of age. **b** G51D cases two (patient III, 2) and three (patient IV, 2), Case two is the parent of case three. A sibling and two members of the previous generation of case two were diagnosed with PD without dementia (*grey*). **c** *SNCA* duplication case (patient III, 3), The father of the duplication case, paternal grandmother and two paternal great-aunts suffered from PD without documented dementia. Her paternal cousin was diagnosed with possible FTDP-17. The H50Q case did not have a family history of Parkinsonism

The index case’s sister (patient II:2), who is alive and is now 49 years old, presented with resting tremor of the left hand at age 40. In the first 5 years, her parkinsonism was slowly progressive but was well controlled by levodopa therapy, with occasional reports of end-of-dose wearing-off and dyskinesia. At age 47, she developed urinary urgency, incontinence and postural hypotension. She also started to fall and became confused with cognitive decline and visual hallucinations. She is now severely debilitated by memory impairment, disorientation, marked akinetic rigidity and urinary incontinence.

### G51D case two: family two, patient III: 2 Fig. 1b

At age 69, this British Caucasian presented with resting tremor of the right hand, anxiety and depression. Examination revealed hypomimia, micrographia, bilateral resting hand tremor, asymmetrical bradykinesia and rigidity with parkinsonian gait and reduced arm swing. Hyperreflexia and mute plantar responses were noted. Initial examination confirmed a normal range of eye movements and normal saccades. Postural hypotension with orthostatic dizziness was documented and urinary urgency developed two years later. There was a good initial levodopa response, which waned after 3 years. At age 71 vivid dreams followed by bizarre behaviour, were reported by the spouse. Confusion and disorientation were frequent and disturbing, persistent visual hallucinations and paranoid delusions were problematic despite reducing the dosage of Ropinirole. A vertical supranuclear gaze palsy with very restricted upgaze and blepharospasm was noted. Rivastigmine was started which led to some improvements in clarity of thought, and fewer visual hallucinations. At age 73, the patient was withdrawn, emotionally labile and had minimal speech output. Episodes of confusion and wandering at night continued, the patient was dependent for all care and was incontinent of urine. In the last 2 years of life, severe dysphagia developed, there was no speech output, spasticity was marked, recurrent chest infections occurred, the patient was confined to bed and died at age 75 after a disease duration of 6 years.

A sibling (patient III: 4) also developed parkinsonism followed by dementia in the fourth decade of life. Clinical deterioration was rapid and death was reported within several years. Medical records and brain tissue were not available for examination.

### G51D case three: family two, patient IV: 2 Fig. 1b

At age 46, this British Caucasian, who was the child of case two, presented with several months history of resting tremor of the right hand and depressed mood. A diagnosis of Parkinson’s disease was made and response to levodopa was good. At age 48, visual hallucinations developed, which did not improve despite withdrawal of dopamine agonist and monoamine oxidase B inhibitor. In the following year, word-finding difficulties were experienced with evidence of perseveration and frontal executive impairment. Motor symptoms and mobility began to deteriorate significantly and falls became a feature. By the age of 50 cognition was profoundly affected with markedly reduced speech production. Examination revealed hypomimia, normal saccadic and pursuit eye movements, bilateral resting hand tremor, bradykinesia, cogwheel rigidity and difficulty copying interlocking pentagons. There were frontal release signs including marked bilateral grasp reflex. Neuropsychometric assessment confirmed frontal and subcortical cognitive impairment. Single-photon emission computerised tomography (SPECT) scan was abnormal with signal reduction in the posterior cortical regions which was compatible with the clinical manifestation of cognitive impairment. At age 51, there was prominent behavioural disturbance, anxiety, irritability and persistent visual hallucinations. The patient become non-communicative, severely dysphagic and was incontinent of urine. Nursing home care was required and death occurred the following year aged 52 after a disease duration of 6 years.

### H50Q mutation case

As previously reported [5], this British Caucasian female developed right hand tremor at age 71 with sustained benefit from levodopa therapy. By age 80 she had marked parkinsonism. She complained of mild memory impairment. Examination revealed tongue tremor, moderate bilateral resting hand tremor, bradykinesia, cogwheel rigidity, hyperreflexia and postural instability. At age 82, there was one report of confusion and hallucination which resolved after withdrawal of bromocriptine. There was no prominent motor fluctuation, cognitive impairment, behavioural change or autonomic dysfunction. She died at age 83 after a disease duration of 12 years. There was no family history of any neurological disorder.

### SNCA duplication case: patient III: 3 Fig. 1c

The details of this case were previously published [12]. This British Caucasian female had longstanding extreme anxiety, panic disorders, hallucinations and a history of seizures. At age 38, she developed tremor of the right hand and the tongue, cervical dystonia, blepharospasm and falls. There was good initial levodopa response, lasting 8 years. At age 47, the most prominent features were obsessional behaviour, poor self-care and a profound increase in appetite, particularly for sweet food. She was diagnosed clinically as having possible frontotemporal dementia with parkinsonism-17 (FTDP-17) but subsequent genetic analysis did not reveal any *MAPT* mutation. Mini mental state examination (MMSE) score was 24/28 excluding a writing task, with most points being lost on attention. Examination showed normal eye movements, hypomimia, jaw tremor, asymmetrical resting tremor, bradykinesia, rigidity, parkinsonian gait with reduced arm swing, hyperreflexia and extensor plantar responses. Prominent frontal impairment was evident with bilateral grasp reflex, magnetic behaviour, perseveration on clapping task and motor recklessness. She also developed postural hypotension and autonomic function testing confirmed cardiovascular dysautonomia. Neuropsychometric findings were compatible with frontal and temporal impairments. She became bedbound and died at age 49 after a disease duration of 12 years. Her father, paternal cousin, paternal grandmother and two paternal great aunts all had a clinical diagnosis of PD, dementia or FTDP-17.

### Neuropathological data

The neuropathological features of all cases are summarised in Table 3.

**Fig. 2:**
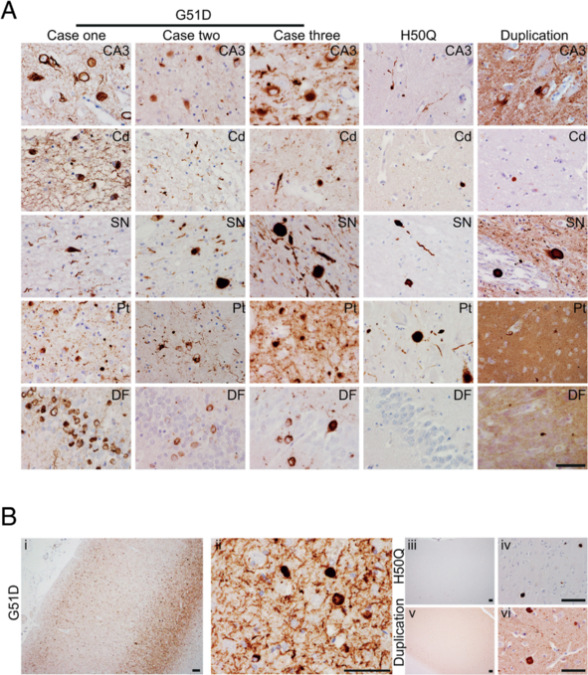
α-Synuclein pathology. **a** Representative microscopy images of paraffin-embedded human brain tissue show abundant neuronal and neuritic α-synuclein pathology in three G51D cases compared to the H50Q mutation and *SNCA* duplication cases stained for α-synuclein protein. High magnification images from CA3, caudate (*Cd*), substantia nigra (*SN*), putamen (*Pt*) and dentate fascia (*DF*). **b** Distinctive neocortical ‘tramline’ deposition of α-synuclein is only detected in G51D cases (*i*, *ii*), shown in representative low (*i*) and high (*ii*) magnification images of the entorhinal cortex, while in H50Q (*iii*, *iv*) and *SNCA* duplication (*v*, *vi*) α-synuclein deposition was detected only in the deep cortical layers (*iv*, *vi*). Scale bars represent 50 μm

In our previous paper we analysed the neuropathology of G51D case one. Here we have compared case one with two further G51D cases from an independent kindred to assess the consistency of the neuropathological features associated with this mutation. The semi-quantitative assessment of regional neuronal loss and both neuronal and glial α-synuclein pathology is presented in Table 2, providing the range of pathological change seen in the three cases. All three G51D mutation cases shared the neuropathological hallmarks which defined case one. There was widespread neuronal and neuritic α-synuclein pathology in all three cases: this included involvement of the neocortex, in addition to the striatum, limbic and brainstem regions. Representative images of these findings are shown in the CA3, caudate (Cd), substantia nigra (SN), putamen (Pt) and dentate fascia (DF) in Fig. 2a. We previously described the varying morphology of the neuronal α-synuclein cytoplasmic inclusions (annular, crescentic, globular, diffuse and neurofibrillary tangle-like). All three cases displayed the same spectrum of inclusion types and in similar distribution, although with some variation in severity. In all cases sparse GCI-like oligodendroglial inclusions were present in the white matter. These were most readily identified in the subcortical white matter, pontine base and cerebellar hemispheric white matter in all cases (Fig. 5a). In addition there were very occasional α-synuclein positive coiled body-like glial inclusions as seen in cases of PD [13]. Some case-to-case variation was observed. In all three cases, the hippocampus showed marked α-synuclein pathology, although the degree of neuronal loss in the CA2-CA3 region was variable between the cases, being most severe in cases one and two. The neocortex showed a similar degree of nerve cell loss in cases one and two where the temporal, cingulate and insular cortices were most severely affected while cortical neuronal loss in case three was no more than mild in any region. In all cases the α-synuclein pathology was most severe in the superficial and deep cortical laminae (Fig. 2b). Balloon neurons, identified by αB-crystallin immunohistochemistry, were quite numerous predominantly in the deep layers of the neocortex, particularly in the frontal, temporal, cingulate and insular cortex (data not shown). Balloon neurons were immunoreactive for α-synuclein and showed weak immunopositivity for p62 and ubiquitin, staining for tau was negative. No argyrophilic grains were identified in the hippocampus using immunohistochemical staining for p62 and tau. There was no Aβ deposition in any of the cases. TDP-43 and tau pathology are described in detail below.

**Table 2:** Summary of neuropathological findings in three cases of G51D mutation

	Neuronal loss	Neuronal α-synuclein pathology					Oligodendroglial α-synuclein inclusions
		Annular or crescent	Globular	Diffuse	NFT-like	Threads	
Cortex							
Frontal	+	++	+/++	+	- /+	++	−/+
Motor	- /+	++	+/++	+	- /+	++/+++	- /+
Temporal	+/+++	+++	+/++	+/++	- /+	+++	- /+
Parietal	+	+/+++	++	+	- /+	++/+++	-
Occipital	-	+	- /++	- /+	- /+	- /+	- /+
Cingulate	+/+++	+++	++	+/++	- /+	+++	-
Insular	+/+++	+++	+/+++	+/++	- /+	+++	-
Sub-cortical white matter							
Frontal	N/A	N/A	N/A	N/A	N/A	+	+
Motor	N/A	N/A	N/A	N/A	N/A	+	+/++
Temporal	N/A	N/A	N/A	N/A	N/A	+	+
Parietal	N/A	N/A	N/A	N/A	N/A	+	+
Occipital	N/A	N/A	N/A	N/A	N/A	+	- /+
Cingulate	N/A	N/A	N/A	N/A	N/A	+	+
Internal capsule	N/A	N/A	N/A	N/A	N/A	+/++	+
External capsule	N/A	N/A	N/A	N/A	N/A	++	+
Amygdala	−/+++	+/++	++/+++	+/++	-	+++	- /+
Hippocampus							
Dentate fascia	-	++/+++	+	+	-	+/++	-
CA4	−/++	+/++	+/++	+	- /+	++/+++	- /+
CA3	−/+++	- /+	+/++	−/+++	-	++/+++	-
CA2	+/+++	-	−/++	−/+++	-	++/+++	-
CA1	−/++	+/+++	+/++	+/++	+	++/+++	-
Subiculum	- /+	+/++	+/++	+/++	- /+	++/+++	-
Entorhinal cortex	−/++	+++	+/++	+/++	- /+	++/+++	-
Transentorhinal cortex	+/++	++/+++	+/+++	+/++	- /+	++/+++	-
Caudate	+	- /+	+/+++	++	- /+	++/+++	-
Putamen	- /+	−/++	+/+++	++/+++	- /+	++/+++	-
Globus pallidus	- /+	−/++	- /+	-	-	+	- /+
Thalamus	-	-	−/+	−/++	-	−/++	- /+
Subthalamic nucleus	-	-	-	- /+	-	−/++	- /+
Red nucleus	-	-	-	-	-	+	+
III^rd^ nerve nucleus	-	- /+	+/++	+/+++	-	+/+++	-
Substantia nigra	+++	-	−/++	−/+++	- /+	++/+++	- /+
Locus coeruleus	++/+++	-	−/++	−/+++	-	++/+++	-
Pontine nuclei	-	-	-	+	- /+	+	- /+
Pontine base white matter	N/A	N/A	N/A	N/A	N/A	+/++	+/++
Dorsal motor nucleus of vagus	+/+++	-	−/+	−/+++	−/+	++/+++	-
Twelfth nerve nucleus	-	-	-	-	-	+	-
Inferior olive	−/+	-	-	−/++	-	+	-
Cerebellar hemisphere Purkinje cells	+/++	-	-	-	-	-	N/A
Cerebellar hemisphere white matter	N/A	N/A	N/A	N/A	N/A	+/++	+/++
Dentate nucleus	-	-	-	-	-	-	-

The range of scores is provided

Oligodendroglial α-synuclein: cytoplasmic inclusions usually with similar morphology to glial cytoplasmic inclusions of MSA, less frequently resembling coiled bodies

*N/A* not applicable, *NFT* Neurofibrillary tangle

In comparison, the H50Q and the *SNCA* duplication cases had less severe α-synuclein pathology, with LB or LN morphology and a distribution typical of PD (Table 3, Fig. 2a). Furthermore, neither case was observed to have α-synuclein inclusions resembling the annular, crescent, or NFT-like pathology of the G51D cases. Neuronal loss was severe in the SN and moderate in the locus coeruleus and in both sites LBs and LNs were present. No neocortical neuronal loss was identified. Neocortical inclusions with features of cortical LBs were present in only moderate numbers and were most prominent in the deep cortical laminae in contrast to the pattern observed in the G51D cases (Fig. 2b). Lewy pathology corresponded to Braak stage 6 in both the *SNCA* duplication and the H50Q cases. GCI-like inclusions were not present in either case although the duplication case did have rare coiled body-like inclusions in the cerebellar and cerebral hemispheric subcortical white matter (Fig. 5b). There was no TDP-43 pathology in the hippocampus, amygdala or striatum in either case. Limited Aβ deposition with sparse diffuse deposits in the temporal cortex and tau neurofibrillary tangle pathology corresponding to Braak and Braak stage I were observed in the *SNCA* duplication case. In the H50Q case numerous diffuse and sparse mature Aβ cortical deposits were present, while neurofibrillary tangle pathology corresponded to Braak and Braak stage III and also involved the DF where there were sparse neurofibrillary tangles. Argyrophilic grains were not observed.

**Table 3:** Summary of neuropathological findings

Pathology	Case 1 (G51D)	Case 2 (G51D)	Case 3 (G51D)	H50Q	SNCA duplication
Cortical neuronal loss	Widespread. Severe in temporal and insular, moderate in cingulate.	Widespread. Severe in cingulate, moderate in temporal and insular.	Widespread mild	None identified	None identified
Hippocampal neuronal loss	CA2/3 predominant	CA2/3 predominant	CA2 mild	None identified	None identified
Caudate neuronal loss	Mild	Mild	Mild	None identified	None Identified
Brain stem neuronal loss	Substantia nigra, locus coeruleus and dorsal motor nucleus of vagus	Substantia nigra, locus coeruleus and dorsal motor nucleus of vagus	Substantia nigra, locus coeruleus and dorsal motor nucleus of vagus	Substantia nigra^b^	Substantia nigra, locus coeruleus and dorsal motor nucleus of vagus
Neuronal α-synuclein pathology	Annular, crescentic, globular, diffuse, NFT-like. Widespread with severe cortical involvement	Annular, crescentic, globular, diffuse, NFT-like. Widespread with severe cortical involvement	Annular, crescentic, globular, diffuse, NFT-like. Widespread with severe cortical involvement	PD type, Braak stage 6	PD type, Braak stage 6
Glial α-synuclein pathology	GCI-like, rarely coiled body type	GCI-like, rarely coiled body type	GCI-like, rarely coiled body type	Absent	Sparse coiled-body type
Phosphorylated tau Braak and Braak stage	II^a^	II^a^	II^a^	III^a^	I
Aβ deposition	Absent	Absent	Absent	Frequent diffuse and sparse mature cortical deposits	Sparse diffuse neocortical deposits
TDP-43 pathology	Hippocampus, amygdala, striatum	Hippocampus, amygdala, rare in striatum	Absent	Absent	Absent

*PD* Parkinson’s disease, *NFT* neurofibrillary tangle

^a^ = dentate fascia also affected

^b^ = locus coeruleus and dorsal motor nucleus of vagus not represented in available sections

### Conformation and phosphorylation of α-synuclein

**Fig. 3:**
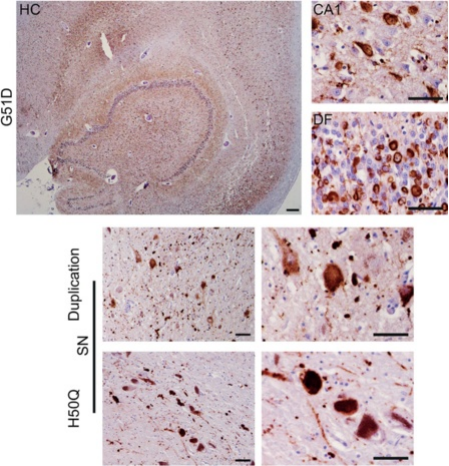
5G4 α-synuclein. The 5G4 α-synuclein antibody has high affinity for high molecular weight α-synuclein oligomers with lesser affinity for fibrils and low affinity for monomeric α-synuclein. Representative images show 5G4 positive α-synuclein accumulation in areas of severe inclusion burden in G51D (*HC*, hippocampus, *CA1*, cornu ammonis 1, DF, dentate fascia) *SNCA* duplication and H50Q (SN, substantia nigra). Scale bars represent 50 μm

We analysed the morphology and phosphorylation state of α-synuclein within inclusions in order to predict the conformation of the protein. The 5G4 α-synuclein antibody was used as it specifically detects high molecular weight α-synuclein oligomers which are nitrated and have β-sheet conformation, with less binding to α-synuclein fibrils and none to monomeric α-synuclein [14, 15] (Fig. 3). We observed 5G4 immunoreactivity in all inclusion types in the G51D, H50Q and *SNCA* duplication cases.

**Fig. 4:**
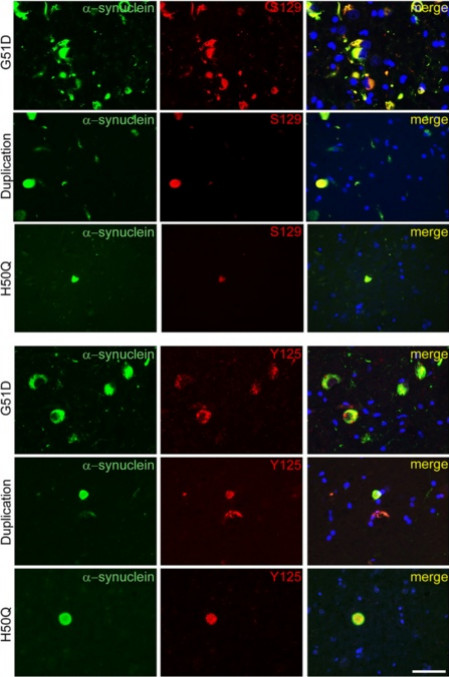
Phosphorylated α-synuclein. Neuronal inclusions in all cases are shown to be immunoreactive for both pro-fibrillar S129 α-synuclein and pro-oligomeric Y125 α-synuclein. Neuritic α-synuclein in the duplication and H50Q cases showed less immunoreactivity of both phospho-α-synuclein epitopes. Representative double immunofluorescence images of mutation cases stained for total α-synuclein (*green*) and phospho-α-synuclein (S129/Y125) (*red*) shown in regions of highest pathology for each case: G51D hippocampus, duplication entorhinal cortex and H50Q SN. Scale bar represents 50 μm

We have previously shown that α-synuclein inclusions in case one are phosphorylated at both the S129 and Y125 epitopes, which is of interest as phosphorylation at the S129 epitope is believed to promote-aggregation into fibrils while Y125 phosphorylation is suggested to result in oligomer formation [16–18]. On investigation of phosphorylation epitopes S129 and Y125 using specific antibodies, we observed that in both the H50Q and the *SNCA* duplication cases only LB were positive for phosphorylated α-synuclein, while LN were seldom immunoreactive indicating low levels of phosphorylated α-synuclein. In contrast in the G51D cases all neuronal and neuritic inclusions were strongly labelled with the antibodies recognising α-synuclein phosphorylated at both the S129 and Y125 epitopes (Fig. 4).

### Inclusions are ubiquitin and P62 positive

Ubiquitin is a small molecule which can become covalently bound to proteins in an event called ubiquitination which is believed to signal for degradation of that protein via the ubiquitin-proteosomal system [19]. P62 recognises ubiquitinated proteins during autophagy and it has been shown that levels of p62 tend to inversely correlate with clearance of aggregated proteins via autophagy [20]. Double immunofluorescence microscopy for ubiquitin (Additional file 1A) or p62 (Additional file 1B) with α-synuclein showed that neuronal and neuritic α-synuclein inclusions in all cases were ubiquitinated. Co-localisation of p62 with α-synuclein was observed in many neuronal inclusions including Lewy bodies but was less prominent in neuritic inclusions.

### GCI-like inclusions occur only in G51D not H50Q or SNCA duplication cases

**Fig. 5:**
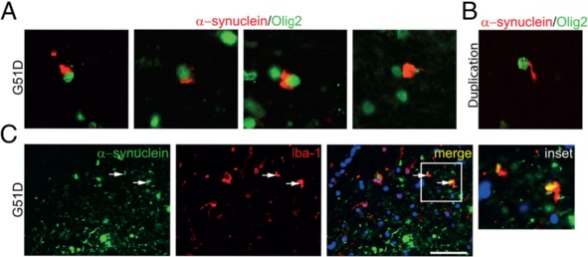
Glial pathology. **a** The variable morphology of GCI-like inclusions in all three G51D cases is shown in representative images in which α-synuclein (*red*) is detected within oligodendrocytes (Olig2, *green*). **b** Rare coiled body-like inclusions of α-synuclein (*red*) are detected within oligodendrocytes (Olig2, *green*) within the cerebellar white matter of the duplication case. **c** On rare occasions α-synuclein (*green*) was confirmed to be present within microglia (Iba-1, red) in G51D cases (*arrows*) shown at high magnification (inset). Scale bar represents 50 μm

In all three G51D mutation cases α-synuclein GCI-like inclusions were detected. These were confirmed as being within oligodendrocytes by double immunofluorescence with the oligodendrocyte marker Olig2 and were detected particularly in the sub-cortical white matter, cerebellar white matter and pons. GCI-like inclusions were observed to be of juxtanuclear conical, rod shaped or globular morphology (Fig. 5a).

The same double immunofluorescence technique did not reveal any oligodendroglial α-synuclein inclusions in the H50Q case. Sparse, elongated α-synuclein positive coiled body-like inclusions within oligodendrocytes of the cerebellar white matter were seen in the *SNCA* duplication case (Fig. 5b).

We observed rare instances in all G51D cases, in which α-synuclein pathology co-localised with microglia as detected using the microglial marker Iba-1 (Fig. 5c, arrows and inset). Co-localisation was confirmed by confocal analysis. There was gliosis in affected regions of both the H50Q and the duplication case, but α-synuclein co-localisation with microglia could not be identified. None of the cases were found to have α-synuclein immunoreactive inclusions within astrocytes (data not shown).

### Phospho-tau and α-synuclein co-localise in a subset of neuronal inclusions in G51D cases, and very rarely in the duplication case and the H50Q case

Tau pathology was considered to correspond to Braak and Braak stage II in all G51D cases, although it was noted that there were also sparse to moderate numbers of neurofibrillary tangles in the granule cells of the DF. Argyrophilic grains were not found in any of the cases.

In our previous study we showed that a proportion of phospho-tau inclusions co-localised with a subset of α-synuclein inclusions within neurons. Therefore, we were interested to determine whether this is a consistent finding in G51D cases two and three and to determine whether this might also be a feature of the H50Q and *SNCA* duplication cases, which had Braak and Braak stages III and I tau pathology, respectively. We used double immunofluorescence of phospho-tau (AT8) with α-synuclein and in each case examined regions of the hippocampus and entorhinal cortex in which phospho-tau pathology was most severe.

**Fig. 6:**
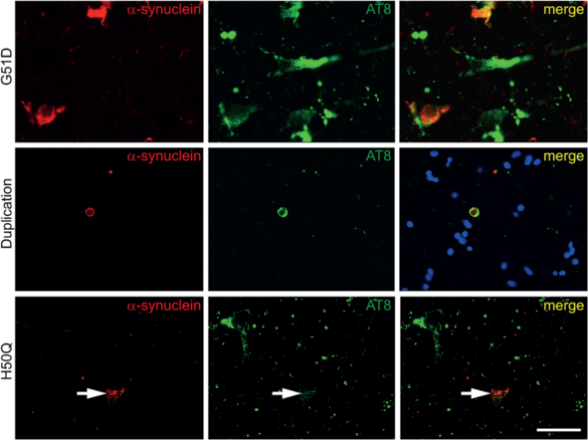
Tau pathology. Double immunofluorescence images of phospho-tau (AT8, *green*) with α-synuclein (*red*) shows co-localisation in a subset of inclusions in G51D cases (shown in CA1) and very rarely in the duplication case (subiculum). Rare examples of sparse diffuse granular co-localisation was observed in the subiculum of the H50Q case (*arrows*). Scale bar represents 50 μm

In keeping with our previous findings, a subset of the α-synuclein inclusions was found to co-localise with tau inclusions in the hippocampus. Whether this subtle association is linked with the biology of the G51D mutant α-synuclein is unclear. In contrast, in the H50Q case, in which tau pathology corresponding to Braak and Braak III with scarce hippocampal or entorhinal α-synuclein inclusions, co-localisation of α-synuclein with tau inclusions was limited to rare cells containing fine granular cytoplasmic structures (Fig. 6, arrows). In the duplication case co-localisation events, although robust, were very rare (Fig. 6) some partial co-localisation of α-synuclein with rare neuritic tau was also detected.

### A subset of G51D α-synuclein inclusions is also immunoreactive for TDP-43

**Fig. 7:**
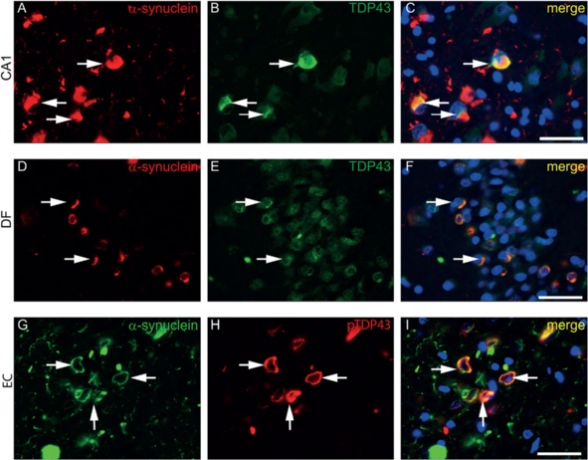
TDP-43 pathology in G51D cases. Double immunofluorescence images show co-localisation of a moderate number of α-synuclein inclusions with TDP-43 (**a**-**f**) or pTDP-43 (**g**-**i**). Representative double immunofluorescence images of α-synuclein (*red*) with TDP-43 (*green*) indicate that TDP-43 inclusions in the CA and entorhinal cortex (EC) co-localise with a subset of α-synuclein inclusions, these events are more rare in the DF (*arrows*). Scale bar represents 50 μm

TDP-43 positive intraneuronal cytoplasmic inclusions were a feature in the hippocampus, amygdala and striatum of G51D case one and case two (Fig. 7) but were absent from case three and could not therefore be considered a consistent feature of the disease (Table 3).

By use of double immunofluorescence for α-synuclein with TDP-43 or phospho-TDP-43, we observed that a subset of TDP-43 inclusions in the CA regions, entorhinal cortex and DF of cases one and two co-localised with α-synuclein inclusions. Representative images of co-localisation of TDP-43 and α-synuclein inclusions are shown in Fig. 7a-f. All TDP-43 inclusions which co-localised with α-synuclein inclusions in G51D cases were also immunoreactive for pTDP-43, as shown in the entorhinal cortex in Fig. 7, g-i. TDP-43 pathology was not detected in either the H50Q or the duplication case.

## Discussion

We have established that consistent clinical and neuropathological features which resemble both those of PD and MSA characterise three G51D *SNCA* mutation cases. The three G51D *SNCA* mutation cases described in this study shared a stereotyped constellation of parkinsonian features with variable levodopa response, dementia, persistent visual hallucinations and autonomic dysfunction. All G51D cases had consistent neuropathological hallmarks which resembled both PD and MSA. These included widespread cortical and subcortical neuronal α-synuclein inclusions together with small numbers of GCI-like inclusions in oligodendrocytes. The principal clinical phenotype of the duplication case bore some similarity to the G51D cases; parkinsonism, dementia and autonomic symptoms were all features. By comparison, the H50Q case had a clinical phenotype consistent with idiopathic PD. Unlike the G51D cases, the neuropathology of the duplication and H50Q cases closely resembled idiopathic PD and GCI-like inclusions were not found in either case.

Predominant frontal release signs, executive dysfunction, perseveration, emotional lability and marked behavioural changes were observed in G51D cases two and three and in the *SNCA* duplication case. All three G51D cases had a good initial levodopa response, but the therapeutic efficacy was transient in cases two and three in which disease progression was rapid. Age of onset in G51D cases was variable, ranging from 19 (case one) to 69 (case two). There appears to be interfamilial variability in the temporal course of disease progression. The second family (Fig. 1b) had a much more rapid deterioration and both cases died only 6 years after symptom onset. Both had an early onset of cognitive decline and loss of levodopa benefit. Significant motor fluctuation was only observed in the first family (Fig. 1a) in which the phenotype resembled that of a monogenetic parkinsonism such as parkin disease, with sustained levodopa response and longer disease duration spanning at least a decade [21]. In both families, depression and anxiety were early features that accompanied the onset of motor symptoms, followed by vivid dreams, intermittent disorientation, word-finding difficulties signifying memory impairment and paranoid delusions. Visual hallucinations were often triggered by a small increase in the dose of dopaminergic medications and would initially be amenable to the adjustment of anti-parkinsonian medications or the introduction of a cholinesterase inhibitor. In more advanced stages of disease, visual hallucinations and behavioural changes became persistent and refractory, eventually dominating the clinical picture along with severe akinetic rigidity and bulbar dysfunction. Dysautonomia including symptomatic postural hypotension, urinary frequency and urge incontinence occurred after the onset of parkinsonism, but was a relatively early feature in G51D *SNCA* families when compared to idiopathic Parkinson’s disease. In both families, the constellation of pathological reflexes, extensor plantar responses and autonomic dysfunction resembles the clinical phenotype of multiple system atrophy, specifically MSA with predominant parkinsonism (MSA-P), but the significant features of dementia with visual hallucinations are a useful pointer to set G51D *SNCA* mutation apart [22]. G51D mutation case two also had some features of a progressive supranuclear palsy phenotype, with unequivocal vertical supranuclear gaze palsy, blepharospasm and emotional lability.

Review of the clinical features reported in the literature in cases of *SNCA* duplication shows a phenotype of parkinsonism, frequently associated with dementia. Autonomic symptoms were observed in 50 % of cases analysed [23]. Case-to-case variability has been reported in relation to age at onset, levodopa responsiveness and motor fluctuations [24, 25]. In our duplication case [12], the clinical syndrome was compatible with frontotemporal dementia with parkinsonism, prompting a tentative clinical diagnosis of FTDP-17 prior to genetic analysis (Fig. 1c). Cervical dystonia, blepharospasm and pyramidal signs were among the other atypical features observed in this case. In contrast, the H50Q *SNCA* mutation case resembled late-onset idiopathic Parkinson’s disease with slow disease progression, sustained levodopa response and the absence of significant dysautonomia, pyramidal signs or dementia. Despite lack of known family history, this case may have a common ancestor with a further H50Q mutation case which has been described in a family with a history of PD [26], suggesting reduced penetrance rather than *de novo* mutation. The reported case had levodopa-responsive parkinsonism without pyramidal or cerebellar signs and, at time of publication, had most recently scored 23 of 30 in the Montreal cognitive assessment indicating mild cognitive impairment. More extensive characterisation of the clinical phenotype awaits the identification of more cases.

Detailed post-mortem examination of the brains of three G51D cases, one H50Q case and a *SNCA* duplication case was performed. The regional and cellular distribution of pathology was assessed in the G51D mutation cases with semi-quantitative assessment of the pathological changes. Despite some variability between cases in the regional severity of the pathology, cases two and three showed marked similarity with case one, the index case we previously reported [6]. All cases had widespread neuronal α-synuclein pathology with marked neocortical involvement displaying the pattern of severely affected superficial and deep cortical laminae. In dementia with Lewy bodies (DLB) the deeper cortical laminae are affected first with involvement of superficial layers seen only with very advanced disease [27]. In contrast to DLB the G51D mutation cases display marked variability in the morphology of neuronal inclusions with many annular and crescentic inclusions in addition to those with appearances more typical of Lewy bodies. The striking involvement of the striatum with α-synuclein neuronal inclusions and threads in case one was mirrored in case two although case three showed only moderate pathology. The neuropathological features of all G51D cases contrasted with those in the H50Q and *SNCA* duplication cases, both of which had an α-synuclein inclusion distribution pattern of typical idiopathic PD consisting of LB and LN in brain stem, limbic and neocortical regions corresponding to Braak stage 6 disease [28]. In the *SNCA* duplication case rare α-synuclein immunoreactive oligodendroglial coiled body-like inclusions were rarely noted as previously described in PD but no GCI-like inclusions were found in either the H50Q or *SNCA* duplication case.

Cell loss in the CA2-CA3 region of the hippocampus, which was prominent in the index G51D case, varied in severity between cases indicating that this pattern of cell loss is not a constant feature of the disease. TDP-43 pathology has been described in around 7 % of PD cases and 19 % of cases with PD dementia while it is rare in MSA [29, 30]. In keeping with these observations TDP-43 positive inclusions were also found to be inconsistent between cases being a prominent feature in only two of the G51D cases and were absent in both the *SNCA* duplication and H50Q mutation case. Sparse GCI-like oligodendroglial inclusions were observed in the white matter in all cases. This suggests that TDP-43 does not have a pathogenic role in these cases.

It is interesting that the neuropathological features that we initially described in association with G51D *SNCA* mutation [6] are similar to those subsequently described in a case with A53E mutation [7]. The A53E mutation case had similarly abundant α-synuclein pathology of variable morphology in neurons and also displayed GCI-like oligodendroglial inclusions. The striatum was severely affected and they observed a similar ‘tramline’ like deposition of α-synuclein pathology in the deep and superficial layers of the cortex. The authors did note differences, for example they observed only mild cell loss in the CA2-CA3 region. This was of interest as our G51D cases two and three showed milder neuronal loss in these regions and thus had greater similarity to the A53E case than to our initial case. In common with our observations in cases one and two Pasanen and colleagues [7] described TDP-43 inclusions in the DF, amygdala and striatum. In our previous publication we compared the neuropathological features of G51D case one with other *SNCA* mutations [6] (Table 2). The strongest similarities were observed between the G51D case and a reported *SNCA* triplication case as well as A53T mutation cases, which were also reported to show accumulation of GCIs, α-synuclein pathology in the striatum and severe CA2/3 neuronal loss. This evidence supports the concept that different mutations of α-synuclein can modify pathological changes and influence the pathways leading to neuronal or glial protein aggregation. Based on the proposed functions of α-synuclein, several pathomechanisms have been suggested by which α-synuclein may mediate or contribute to cell death including aberration of synaptic signalling [31, 32], mitochondrial dysfunction [33] and loss of chaperone function [34]. Both mutations and post translational modifications including phosphorylation, ubiquitination, nitration and glycosylation [35–38] have been shown to contribute to disease pathogenesis.

We explored the co-localisation of TDP-43 with α-synuclein within inclusions showing that this occurred in a subset of inclusions and this was more common in the CA2-CA3 region than in the DF. Interestingly it was also in these neurons of the CA regions, DF and entorhinal cortex in which we observed co-localisation of α-synuclein with phosphorylated tau in a subset of neurons in all three G51D cases. Although we were unable to investigate this in the current study, this suggests that α-synuclein, tau and TDP-43 pathology could potentially all be present together in a proportion of these neurons. This feature in two of our three G51D cases resembles that of a case of corticobasal degeneration, which was reported to show partial co-localisation of α-synuclein, TDP-43 and tau in inclusions supporting the concept of ‘cross-seeding’ of pathology [39]. The coexistence of tau with α-synuclein in inclusions is not a new observation, tau has long been known to be present in LBs of both PD and Alzheimer’s disease with amygdala LBs, especially in neurons which are particularly vulnerable to tau pathology [40]. Co-localisation of α-synuclein and tau as hybrid oligomeric species may also occur in PD and DLB [41].

The phosphorylation of α-synuclein has been reported, based on *in vitro* studies, to either promote (S129) fibrillisation/aggregation or prevent aggregation and promote oligomerisation (Y125) [16–18]. We have shown that α-synuclein inclusions in G51D cases are frequently phosphorylated at both the S129 and Y125 epitopes. In contrast fewer α-synuclein structures in the H50Q and duplication cases were found to be phosphorylated. This could indicate that the G51D mutation leads to a protein conformation which predisposes to phosphorylation. Furthermore, α-synuclein is reported to be cleaved by cathepsin D at Y125 and phosphorylation of this epitope may prevent lysosomal degradation of α-synuclein [42]. Thus the high degree of phosphorylation and abundance of aggregated α-synuclein, detected using the 5G4 antibody, could suggest that pathological hyperphosphorylation leads to impaired clearance [43], which favours the development of the abundant cellular α-synuclein inclusions characteristic of G51D mutation. We also showed co-localisation of ubiquitin and p62 with α-synuclein in inclusions in all five cases. Both p62 and ubiquitin play important roles inthe proposed mechanisms of α-synuclein proteolysis and our observations point to the necessity for future studies to investigate the role of impaired protein degradation in cases with *SNCA* mutation.

The G51D and H50Q *SNCA* mutations are immediately adjacent in the putative protein loop that results in the hairpin formation of α-synuclein protein [8]. α-Synuclein has been proposed to form tetramers endogenously which resist disease-associated aggregation [44–46], although this proposed structure is a matter of on-going debate [47]. Disruption of the protein loop is believed to impair tetramer formation making mutant α-synuclein monomers more susceptible to oligomerisation and aggregation [8]. It seems likely that each specific mutation of α-synuclein affects the ability of the protein to form fibrillar aggregates to a different degree, resulting in distinct clinical [23] and neuropathological phenotype. This theory is supported by data presented here which shows the distinctly different phenotype of G51D cases compared to the H50Q case despite the fact that the sites of the mutations are immediately adjacent in the putative protein loop region.

The effect of *SNCA* point mutation on α-synuclein aggregation has been a topic of discussion as a factor that may contribute to inclusion formation. In general the insight gained from investigations into *in vitro* disease models has been limited as they do not account for the contribution of factors such as dysfunction of clearance mechanisms [48] and neuroinflammation [49, 50] as in the disease state. The A53T and E46K mutant α-synuclein proteins are reported to aggregate more rapidly [51–53] than the wild-type (WT) protein while the A30P has a more uncertain effect [54, 55]. The G51D and H50Q mutations have also been reported to have variable effects on α-synuclein aggregation. The G51D mutation has been reported to result in decreased aggregation [10, 56] increased oligomer formation and significantly increased cell toxicity in the wake of stressors H_2_0_2_ and MPP+ treatment [57]. While the H50Q mutation has been reported to aggregate into fibrils more rapidly, it formed oligomers less readily than G51D or WT α-synuclein and there was a trend towards increased cell toxicity in response to stressors in culture [57–61]. Neither mutant was shown to result in increased inclusion formation in cultured cells. The A53E mutation has also been reported to cause increased oligomer formation compared to WT protein [58]. The readiness with which the G51D and A53E mutant proteins form oligomers could prove to be relevant in understanding their associated pathology if the oligomeric species are the more toxic forms of the protein [62, 63].

The α-synuclein 5G4 antibody [14, 15] was shown to specifically detect α-synuclein oligomers in a high molecular weight, nitrated, β-sheet conformation and to have lesser affinity for fibrils and not to bind the disordered oligomers or monomers found in synaptic termini [15], We used the 5G4 antibody to demonstrate that the accumulation of α-synuclein in a β-sheet oligomeric conformation is widespread in G51D cases. In the H50Q and duplication cases all neuritic or LB inclusions were immunoreactive for 5G4, indicating that, at the time of death, inclusions in G51D cases are no less aggregated by comparison despite the *in vitro* data suggesting slower aggregation properties conferred by this mutation. Furthermore, we did not detect severe or widespread accumulation of inclusions in the H50Q case compared with the spectrum of pathology in idiopathic PD. If H50Q α-synuclein does aggregate faster than G51D α-synuclein *in vivo*, the short fibrils which it is reported to form [57] may exist only transiently and be cleared by normal mechanisms. Studies in disease cases have shown that in some the neuropathology of the most rapidly aggregating mutant α-synuclein protein, A53T, bears some similarity to that of G51D and A53E mutations [7], including CA2 cell loss, and GCI-like inclusions [64] for review [6]. The neuropathology of the H50Q case bears greater similarity to that of the more slowly aggregating A30P mutant [65], which gives rise to a pathological phenotype resembling sporadic PD [66]. Some cases of *SNCA* duplication have been reported, like G51D, to have GCI-like inclusions,’ tramline’ like cortical deposition of α-synuclein pathology in the deep and superficial layers of the cortex and CA2-CA3 cell loss [67–69]. Although neuronal loss and α-synuclein inclusions were more abundant and widespread in our duplication case than the H50Q case (Table 3), the pathology did not reach the severity of the G51D cases and resembled sporadic PD. Although only a single duplication case was available for this study, our findings indicate that increased α-synuclein expression is not the sole factor which determines the abundance of α-synuclein inclusions and neuronal loss. Altered conformation of the protein due to mutation may impede protein clearance mechanisms thus predisposing to a high load of α-synuclein containing intracellular inclusions.

## Conclusions

Here we have described the spectrum of clinical and neuropathological characteristics of a small series of G51D *SNCA* mutation cases providing information to facilitate the recognition of this clinicopathological entity. Our detailed analysis confirms that clinical features including variable levodopa response, dementia, persistent visual hallucinations and autonomic dysfunction were consistent in all three cases. The neuropathological features of all three G51D cases share characteristics of both PD and MSA these included widespread cortical and subcortical neuronal α-synuclein inclusions together with small numbers of GCI-like inclusions in oligodendrocytes. We have shed light on the differential effects of *SNCA* mutations on neuropathology from which we have gained insight into the biology of pathological α-synuclein. It is vital that we further our understanding of the biology of α-synuclein in disease in order to identify potential pathways and mechanisms which can be targeted for therapeutic intervention.

## Methods

### Clinical data

Medical records, including notes from the general practitioners, letters from hospital specialists and in-patient notes, were retrospectively reviewed by a neurologist with a specialist interest in movement disorders (HL). Clinical symptoms and signs not documented in the notes were considered as absent. Where there was discrepancy in the clinical features described, the neurologists’ record was used.

Consent for research was obtained for all cases included in the study.

### Brain tissue

Three cases with G51D and one case with the H50Q *SNCA* mutation were donated to the Queen Square Brain Bank for Neurological Disorders, UCL Institute of Neurology. The donation protocols had Research Ethics Committee approval and the tissue was stored for research under a license issued by the Human Tissue Authority (No. 12198). Following fixation in 10 % buffered formalin, the right half of the brain was sliced in the coronal plane, examined and blocks were selected for paraffin wax embedding and histology. The *SNCA* duplication case was donated to the Department of Neuropathology, North Bristol NHS Trust, Bristol with consent for research.

Paraffin-embedded sections (8 μm) were stained with haematoxylin and eosin (H&E) and Luxol fast blue/cresyl violet. Immunohistochemistry was performed as previously described [70] using primary antibodies (Additional file 2). Double immunofluorescence was detected using isotype specific anti-rabbit IgG or anti-mouse IgG secondary antibodies conjugated with either Alexa 488 or 568 fluorochromes (1:400) (Life technologies, Paisley, UK) followed by quenching or autofluorescence with 0.1 % Sudan Black/70 % ethanol (Sigma-Aldrich, Dorset, UK) solution for 10 min and mounting under glass coverslips using VECTAshield mounting media with 4’,6-diamidino-2-phenylindole (DAPI) nuclear stain (Vector laboratories, Peterborough, UK). Images were visualised using a confocal fluorescence microscopy (Leica DM5500 B).

### Genetics

Genetic analysis of G51D *SNCA* mutation cases two and three was performed using Sanger sequencing of the *SNCA* gene as previously described [6]. The H50Q case was described by Proukakis et al. [5] and the *SNCA* duplication case was identified using multiplex ligation dependent probe amplification (MLPA) and DNA array analysis, Kara et al. [12].

## Additional files

Additional file 1:
**A, Double immunofluorescence images of ubiquitin (green) with α-synuclein (red) in a representative G51D case (CA3), the duplication case (SN) and the H50Q case (EC).** B, Representative immunofluorescence image of α-synuclein (red) and p62 (green) in G51D (CA1), the duplication case (substantia nigra) and the H50Q case (Temporal cortex). Scale bar represents 50 μm. (TIF 5,615 kb) Additional file 2:
**Antibodies used in study.** (DOCX 16 kb)

## Abbreviations

PD: Parkinson’s disease
MSA: Multiple system atrophy
GCIs: Glial cytoplasmic inclusions
DLB: Dementia with Lewy bodies
LB: Lewy bodies
LN: Lewy neurites
CA: Cornu ammonis
SPECT: Single-photon emission computerised tomography
FTDP-17: Frontotemporal dementia with parkinsonism-17
MMSE: Mini mental state examination
Cd: Caudate
SN: Substantia nigra
Pt: Putamen
DF: Dentate fascia
MSA-P: MSA with predominant parkinsonism
HC: Hippocampus
EC: Entorhinal cortex
