# Supplementary figures and images for: Distinct clinical and neuropathological features of G51D SNCA mutation cases compared with SNCA duplication and H50Q mutation
Source: Mol Neurodegener. 2015 Aug 27;10:41. doi: 10.1186/s13024-015-0038-3 (PMC4549856; doi:10.1186/s13024-015-0038-3)

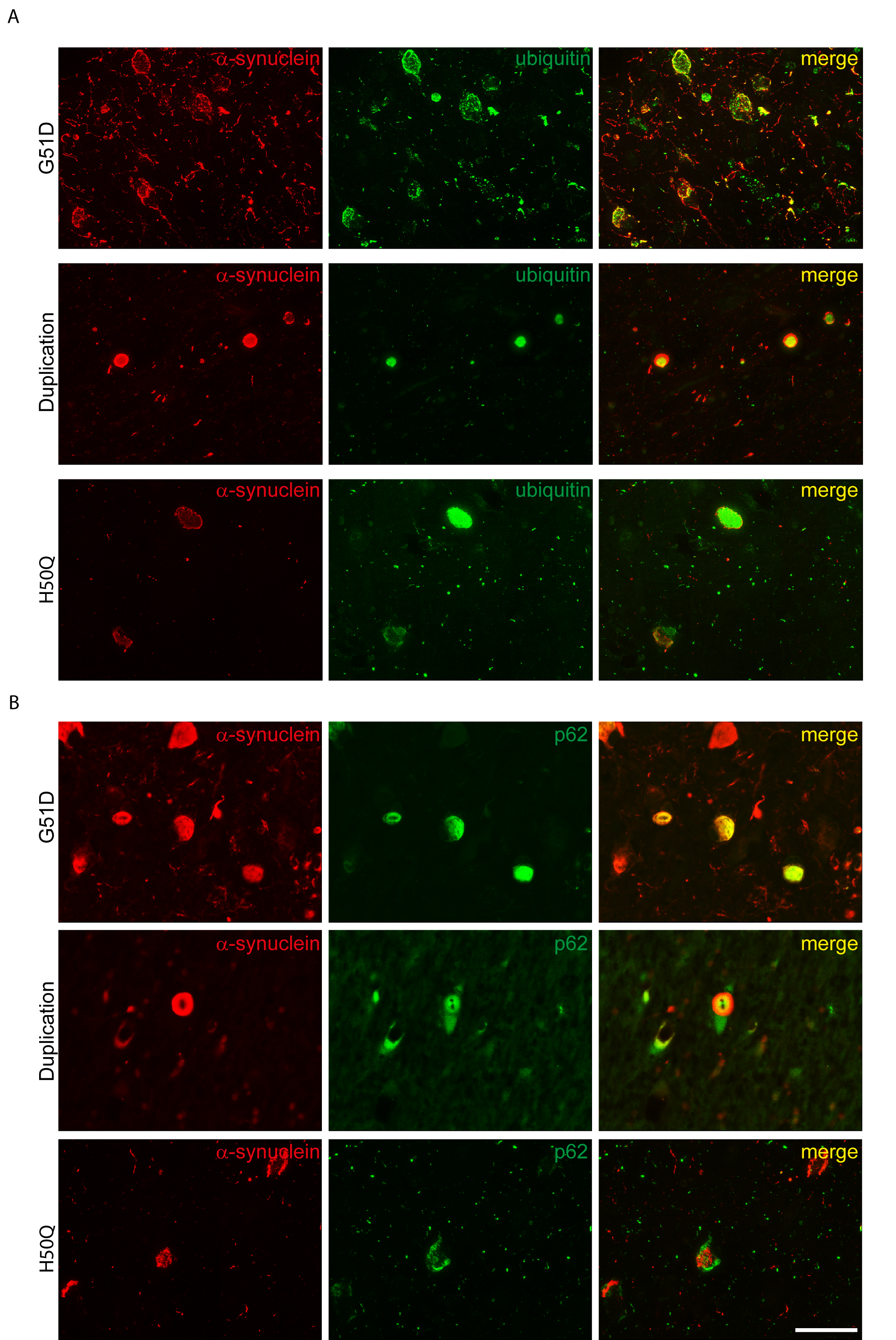

Supplement: Additional file 1: — A, Double immunofluorescence images of ubiquitin (green) with α-synuclein (red) in a representative G51D case (CA3), the duplication case (SN) and the H50Q case (EC). B, Representative immunofluorescence image of α-synuclein (red) and p62 (green) in G51D (CA1), the duplication case (substantia nigra) and the H50Q case (Temporal cortex). Scale bar represents 50 μm. (TIF 5,615 kb) [file 13024_2015_38_MOESM1_ESM.tif]
