# Supplementary material for: Distinct clinical and neuropathological features of G51D SNCA mutation cases compared with SNCA duplication and H50Q mutation
Source: Mol Neurodegener. 2015 Aug 27;10:41. doi: 10.1186/s13024-015-0038-3 (PMC4549856; doi:10.1186/s13024-015-0038-3)
Supplement: Additional file 2: — Antibodies used in study. (DOCX 16 kb) [file 13024_2015_38_MOESM2_ESM.docx]

**Supplementary table 1. Primary antibodies used in the study**

| *Antibody (clone/epitope)* | *Clonality* | *Supplier (catalogue number)* | *Application* | *Dilution* | |
| --- | --- | --- | --- | --- | --- |
| α−synuclein (KM51) | monoclonal | Vector (VP-A106) | IHC/IF | 1:50 | |
| α−synuclein (amino acids 111-131, C-terminal) | polyclonal | Abcam (ab15530) | IF | 1:800 |  |
| α-synuclein (5G4) | Monocloncal | Aj Roboscreen | IHC | 1:100 |  |
| Phospho-α-synuclein (Ser129) | polyclonal | Abcam (ab59264) | IF | 1:100 |  |
| Phospho-α-synuclein (Y125) | polyclonal | Abcam (ab10789) | IF | 1:75 |  |
| Aβ (6F/3D) | monoclonal | DAKO (M0872) | IHC | 1:100 |  |
| AT8 (Ser202/Thr205) | monoclonal | Source Bioscience (90206) | IHC/IF | 1:600 |  |
| α−B-Crystallin (G2JF) | monoclonal | Novocastra (ABCRYS-512) | IHC | 1:300 |  |
| Ubiquitin | polyclonal | DAKO (Z0458) | IHC | 1:200 |  |
| P62 (3/P62 LCK LIGAND) | monoclonal | BD Transduction (610833) | IHC/IF | 1:100 |  |
| TDP-43 (2E2-D3) | monoclonal | Abnova (H00023435-M01) | IHC/IF | 1:2500 |  |
| pTDP43 | Polycloncal | Cosmo Bio Co., LTD | IHC/IF | 1:500 |  |
| GFAP | polyclonal | Dako (Z0334) | IHC/IF | 1:1000 |  |
| Iba-1 | polyclonal | Wako (091-19741) | IF | 1:500 |  |
| Olig-2 | polyclonal | Abcam (ab42453) | IF | 1:500 | |
|  |  |  |  |  | |

IHC, immunohistochemistry; IF, Immunofluorescence
